# Multidimensional synergistic adaptive mechanisms of *Betula luminifera* and its implications for ecological restoration in karst habitats

**DOI:** 10.3389/fpls.2026.1963491

**Published:** 2026-10-28

**Authors:** Duoteng Tang, Shun Zou, Jie Xiao, Tu Feng, Mingchun Liu, Yingdie Yang

**Affiliations:** 1 School of Ecological Engineering, Guizhou University of Engineering Science, Bijie, Guizhou, China; 2 College of Life and Health Science, Kaili University, Kaili, Guizhou, China; 3 Key Laboratory of Bio-Resource and Eco-Environment of Ministry of Education, College of Life Sciences, Sichuan University, Chengdu, China

**Keywords:** *Betula luminifera*, calcareous soil adaptation, carbon economy, ecological restoration, karst habitat, synergistic adaptation

## Abstract

Karst ecosystems impose compound stresses of seasonal drought, calcium-rich substrates, and nutrient poverty, which severely constrain plant survival and ecological restoration. *Betula luminifera*, a native pioneer species of subtropical China, has emerged as a key candidate for restoring such degraded landscapes; however, research on its adaptation mechanisms remains fragmented, with individual physiological traits rarely integrated into a mechanistic framework. To address this gap, this review systematically synthesizes the existing evidence and proposes a carbon-driven synergistic adaptation framework, in which photosynthetic carbon serves as the universal currency, dynamically allocated among growth, resource acquisition, and defense to optimize fitness under compound stress. The framework integrates three interdependent systems: (1) an efficient photosynthetic-water system that manages the carbon-water trade-off and establishes a high-throughput carbon economy; (2) a rhizosphere nutrient-mobilization strategy for overcoming phosphorus limitation that is currently based on comparative rather than species-specific evidence and therefore requires exudate level validation, coupled with efficient internal nutrient cycling; and (3) a coordinated defense system combining antioxidant enzymes and osmotic adjustment to mitigate oxidative stress, with ion compartmentalization and calcium-management strategies (e.g., root sequestration, vacuolar storage) proposed as key components pending molecular validation. These systems are proposed to be coordinated through dynamic carbon allocation, although direct evidence for such allocation in *B. luminifera* is currently lacking. Under water limitation, a tentative priority hierarchy is proposed in which investment in photosynthetic tissues and hydraulic integrity outranks rhizosphere exudation and resource-intensive defense, with exudation activated mainly under phosphorus deficiency. The key contribution of this review is not the enumeration of individual adaptations but the construction of an integrative framework that explains how *B. luminifera* coordinates multiple strategies to overcome the karst stress triangle, explicitly separating direct evidence, comparative evidence, and proposed mechanisms. Because the rhizosphere phosphorus-mobilization pathway and calcium detoxification pathways currently rest on comparative rather than species-specific evidence, molecular validation of root exudation and Ca^2+^ handling is a top priority. Future research should also prioritize long-term field trials, including direct comparisons of monoculture versus mixed-community restoration, to assess the predictive power of the framework for guiding precision restoration and developing stress-tolerant germplasm of *B. luminifera*.

## 1 Introduction

Karst ecosystems, covering approximately 15% of the global land area (Goldscheider et al., 2020), are defined by a core stress triangle of seasonal drought, Ca-rich substrates, and nutrient poverty (Wang et al., 2019). These stresses collectively severely limit vegetation restoration. This limitation is particularly acute in regions like southwestern China, one of the world’s largest contiguous karst zones, where slow soil formation, discontinuous soil cover, and high bedrock permeability synergistically exacerbate water and nutrient deficits (Hongyan et al., 2021; Zhang et al., 2022). Consequently, identifying and utilizing plant species with inherent multi-stress resilience is paramount for effective restoration (Wu et al., 2024; Liu et al., 2025). *Betula luminifera* H. Winkl. exemplifies this resilience, combining a suite of morphological and physiological attributes with proven restoration value.

A tall deciduous tree reaching 20–25 m in height and up to 80 cm in diameter at breast height (DBH), *B. luminifera* is characterized by smooth, reddish-brown bark and glandular-resinous leaves (**Figure 1**). Endemic to subtropical China (e.g., Guizhou, Guangxi, Yunnan, Sichuan, Hubei, Zhejiang) it occurs naturally at elevations of 500–2,500 m, producing hard, fine-grained timber that is widely used for furniture, construction, and papermaking (Li and Zheng, 1979). Ecologically, its drought-tolerant, adaptability to infertile calcareous soils, and vigorous regeneration ability have established it as a pioneer species for rehabilitating karst rocky desertification, degraded mining lands, and barren mountain sites. Despite the breadth of the ecophysiological studies on these attributes, an integrated mechanistic framework remains lacking.

**Figure 1:**
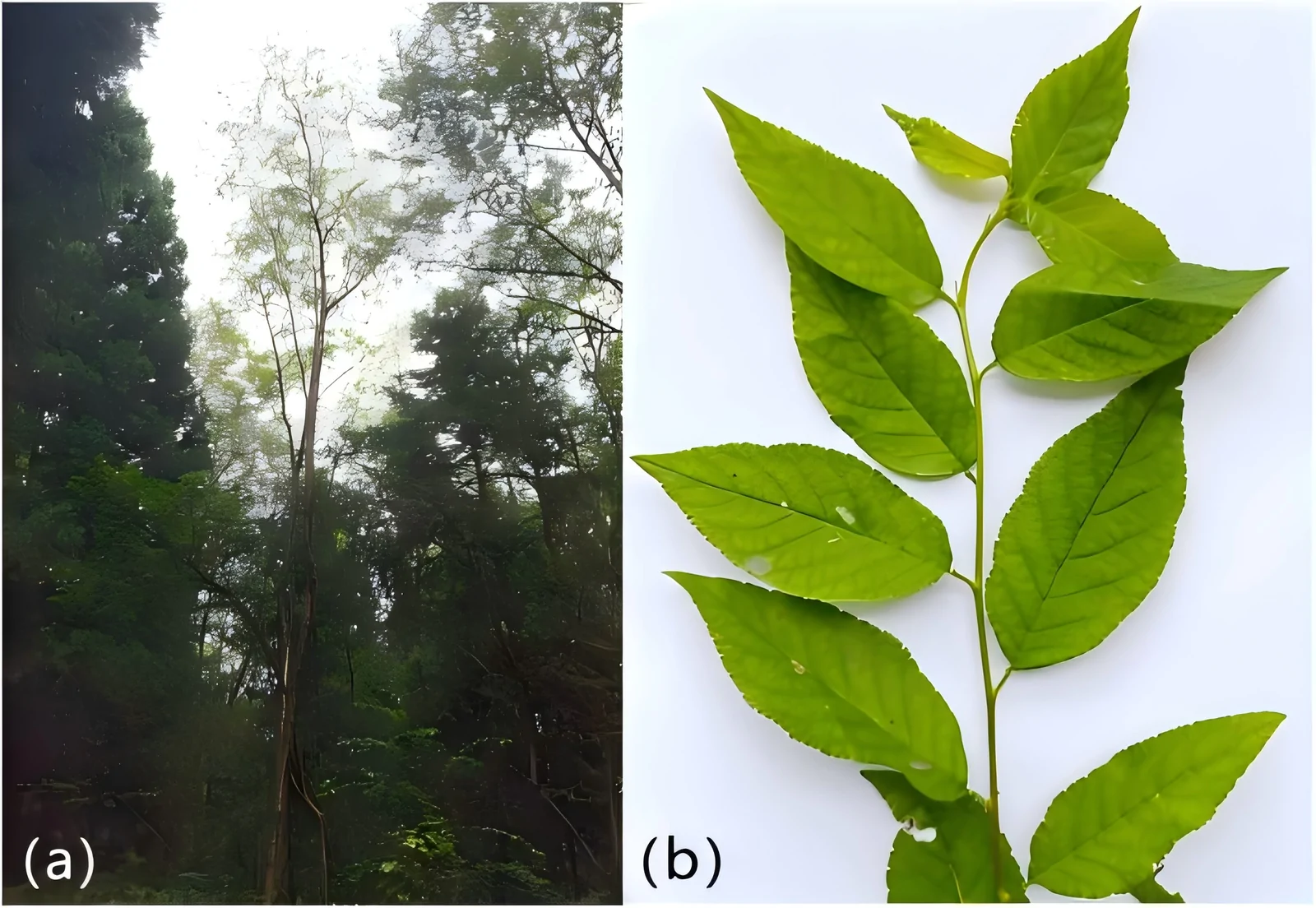
Morphological appearance of *Betula luminifera*. **(A)** Whole-plant habit, **(B)** Leaves and branch. These photographs illustrate species appearance and do not, by themselves, establish morphological adaptation to karst conditions.

Current research on *B. luminifera* remains fragmented. Studies predominantly focus on isolated traits rather than integrated mechanisms. For instance, stand-level biomass accumulation (Ning et al., 2011; Teng et al., 2016) and species-level stress physiology (Hu et al., 2024) have been examined largely in parallel, with little cross-scale synthesis. Moreover, the synergistic effects of multiple stressors are poorly understood. This knowledge gap hinders the full application of *B. luminifera* in ecological restoration (Li et al., 2001; Yang et al., 2024), potentially contributing to suboptimal restoration outcomes in challenging environments.

To address this gap, this review constructs a synergistic framework that integrates the coordinated physiological-ecological strategies enabling *B. luminifera* to optimize carbon capture, resource acquisition, and stress resilience (**Figure 2**). The key innovation is the systematic synthesis of evidence for each sub-system and the delineation of how carbon economy mediates trade-offs and synergies among them. This work moves from the preliminary concept of synergistic adaptation toward an explicitly testable framework, theoretically elucidating system-level adaptive responses and practically guiding restoration in karst and degraded mining areas. Finally, we identify key research gaps, particularly in molecular mechanisms and long-term efficacy under climate change, to highlight future directions for precision restoration and germplasm development.

**Figure 2:**
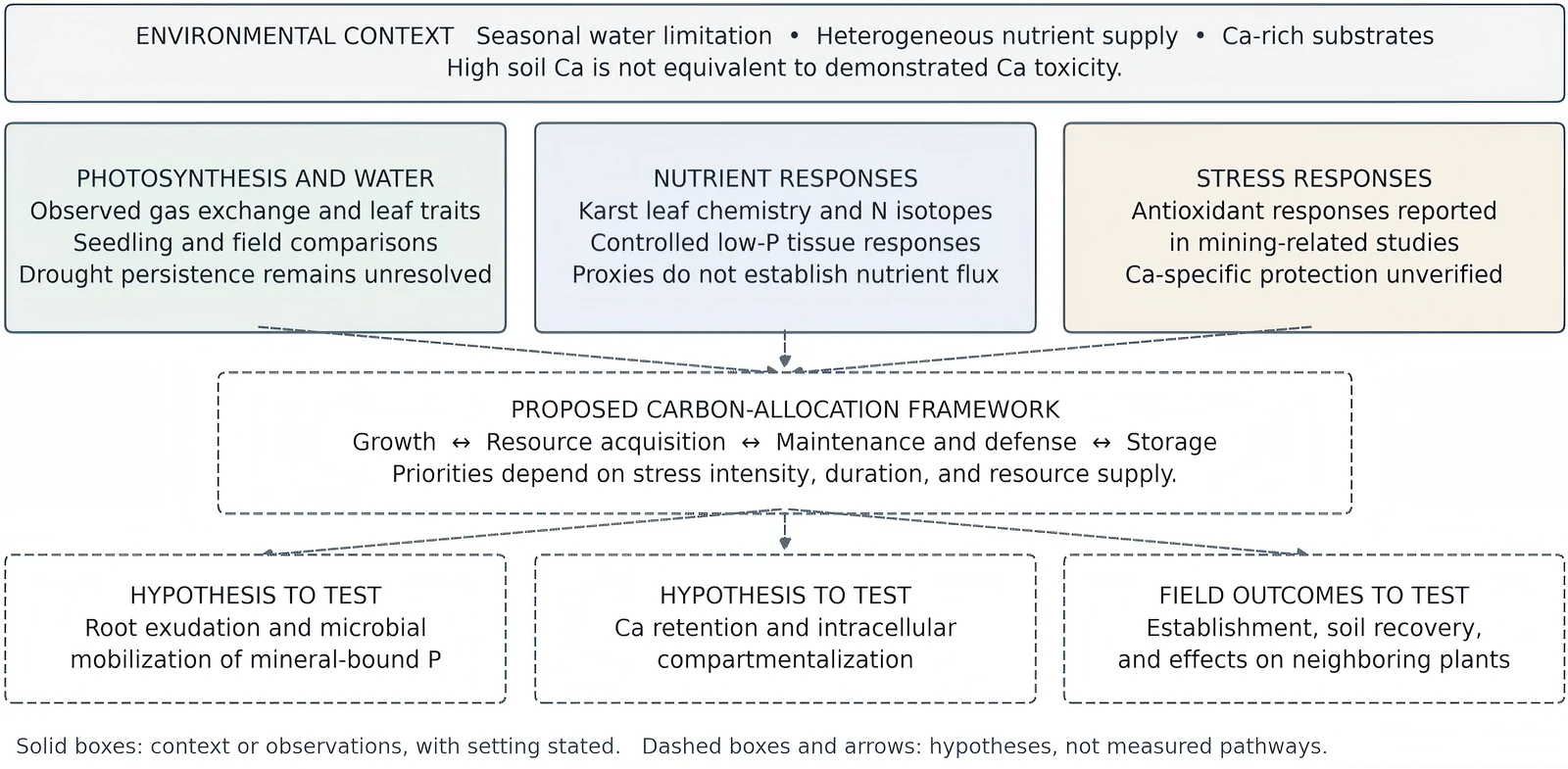
Conceptual framework of the carbon-driven synergistic adaptation of *Betula luminifera* in karst habitats. This conceptual framework illustrates the carbon-driven synergistic adaptation of *B. luminifera* in karst habitats, organized into 5 hierarchical levels: (1) core assumption, photosynthetic carbon as the universal currency for dynamic allocation; (2) three core systems, an efficient photosynthetic-water system (carbon engine), rhizosphere mobilization and nutrient cycling system (nutrient security), and ion-oxidative defense homeostasis system (stress tolerance); (3) system integration, carbon budgeting mediates trade-offs among growth, resource acquisition, and defense; (4) individual plasticity, morphological and physiological adjustments under environmental filtering; and (5) ecological application, translation of individual mechanisms to community-level restoration outcomes. the framework posits that the dynamic allocation of photosynthetic carbon among competing demands is the unifying principle that coordinates the three systems, enabling flexible, context-dependent adaptation to the karst stress triangle. solid boxes indicate mechanisms with direct experimental evidence; dashed boxes indicate proposed mechanisms requiring further validation.

To evaluate the evidence presented at each step of the framework, we adopt the following evidence hierarchy throughout this review. We use “direct evidence” for measurements obtained specifically in *B. luminifera*; “comparative evidence” for findings extrapolated from other *Betula* species, karst plants, or analogous stress responses; and “proposed mechanism” for logical inferences that require direct validation in *B. luminifera*. Accordingly, the proposed carbon allocation framework is presented as a testable hypothesis, not an established mechanism.

## 2 Drought adaptation via an integrated photosynthetic-water system and strategic carbon economy

The adaptation of *B. luminifera* is orchestrated by three synergistic systems. This section elucidates the first and foundational system, namely an integrated photosynthetic-water system. It establishes a high-throughput carbon economy. This system is pivotal because it generates and manages the primary currency for all adaptation, photosynthetic carbon (Fu et al., 2019). By strategically resolving the carbon-water trade-off, it ensures survival under seasonal drought and supplies the resources that fuel the other systems.

The primary challenge to this carbon economy is water. In karst habitats, seasonal drought coupled with rapid soil water loss constitutes a primary constraint (Cao et al., 2020; Ding et al., 2021). Water stress triggers a carbon crisis by forcing stomatal closure, which reduces water loss but also impedes CO_2_ uptake (Cornic, 2000; Tong et al., 2019). Therefore, the challenge is to maximize carbon assimilation under limited water availability. *B. luminifera* achieves this through the synergistic operation of three interconnected mechanisms: (1) a high-efficiency photosynthetic engine, (2) dynamic, plastic water regulation, and (3) pioneer-type carbon investment.

The first mechanism, a high-efficiency photosynthetic engine, is evidenced by *B. luminifera’s* robust carbon assimilation under drought. Its net photosynthetic rate (Pn) is significantly higher than that of conservative karst species such as *Quercus fabri* [a 2.4-fold increase; (Xing et al., 2015)]. This is underpinned by comparatively high carboxylation efficiency (0.05 µmol·m^-2^·s^-1^) and elevated carbonic anhydrase activity (605.19 U·g^-1^), enabling efficient CO_2_ fixation. The species also displays a wide photosynthetic light-response range, with light saturation points of 1,229–1,675 µmol·m^-2^·s^-1^ and compensation points of 19–39 µmol·m^-2^·s^-1^ (Tang, 2015), facilitating carbon gain under variable light (Du et al., 2019). Critically, this system remains productive under stress: in sulfur-iron mining areas, its maximum photosynthetic rate (A_max_) is 2–4 times greater than that of sympatric species (Tang, 2015), underscoring its role as a pioneer reliant on rapid carbon accumulation (Gao et al., 2014; Teng et al., 2016).

The second mechanism, dynamic and plastic water regulation, supports the carbon fixed by the photosynthetic system. First, *B. luminifera* exhibits high stomatal plasticity, with diurnal stomatal conductance (Gs) frequently showing multi-peak patterns (Fan et al., 2010). This allows opportunistic exploitation of transient favorable conditions while minimizing water loss during stress peaks (Wu et al., 2021, 2022b), mitigating midday photosynthetic depression and enhancing daily carbon gain (Yang et al., 2023). Second, it employs an acquisitive, high-transpiration (Tr) strategy. Unlike species employing a conservative water-saving strategy (e.g.,*Q. fabri)*, *B. luminifera* maintains a high Tr to support nutrient transport and cooling, yet its water use efficiency (WUE ≈ 10.27 mmol·mol^-1^) remains competitive with *Q. fabri* (Xing et al., 2015). This is possible because its proportionally higher photosynthetic rate allows it to “dilute” the water cost of carbon gain. Furthermore, *B. luminifera* plastically adjusts Tr in response to soil moisture decline (Cai et al., 2023), suggesting context-dependent water regulation.

The third mechanism, pioneer-type carbon investment, determines how the acquired carbon is ultimately used. *B. luminifera* prioritizes growth and colonization while maintaining baseline stress resilience (Hu et al., 2024; Li et al., 2026). Morphologically, it combines high specific leaf area with high WUE (Yu et al., 2019; Wu et al., 2022b), directing carbon toward photosynthetic tissues. Under drought, it increases its root-to-shoot ratio, a morphological adjustment that enhances water uptake at the expense of aboveground growth (Liu et al., 2023). Physiologically, when facing concurrent stresses, *B. luminifera* adjusts its carbon budget by up-regulating low-metabolic-cost antioxidant enzymes (e.g., SOD, POD) over energetically expensive osmolyte accumulation, a trade-off that prioritizes growth under moderate stress (Jiang, 2024; Zeng et al., 2024).

It should be noted that part of the evidence cited above originates from non-karst habitats, including Sb-contaminated mining areas (Jiang, 2024), sulfur-iron mining sites (Tang, 2015), and nursery experiments (Fan et al., 2010), where the stress combinations differ from those typical of karst. For instance, under acute toxic stress (e.g., Sb contamination in mining habitats), the up-regulation of antioxidant enzymes suggests, but does not prove, a shift of carbon toward defense, as the concurrent growth reduction could equally reflect an overall constrained carbon supply rather than active reallocation. The hypothesized Ca retention and vacuolar sequestration may represent carbon-efficient defense, but this remains a working model awaiting molecular validation (Section 4). Because these toxic-stress observations originate from non-karst, contaminated habitats, their transferability to the karst stress combination (drought, high Ca, and low P) requires direct testing.

In summary, the drought adaptation of *B. luminifera* is the cornerstone of its carbon-driven synergistic framework. The integration of a high-efficiency photosynthetic engine, dynamic water regulation, and growth-prioritizing carbon allocation does more than cope with scarcity; it establishes a high-throughput carbon economy that fuels the entire plant. This robust carbon budget provides the necessary, but not sufficient, currency for investment in the other two synergistic systems: rhizosphere processes for nutrient mobilization (Section 3) and the synthesis of defense compounds (Section 4). Whether this currency is actually spent on those systems depends on a functioning carbon-allocation rule that determines when and how it is spent; a testable priority hierarchy for this rule is developed in Section 5, where the trade-offs among growth, acquisition, and defense are integrated within the framework of carbon as the universal currency.

## 3 Adaptation to nutrient scarcity through rhizosphere mobilization and efficient internal cycling

Nutrient scarcity, particularly of phosphorus (P), is a primary constraint on the growth and survival of *B. luminifera* in karst habitats. Karst soils are rich in exchangeable Ca^2+^ but extremely low in available P (Tang et al., 2021). Under such constraints, plants typically adopt a conservative strategy that prolongs tissue nutrient-retention times and maximizes internal recycling (Liu et al., 2014), or an acquisitive strategy that expands root foraging and enhances rhizosphere mobilization, or both (Gao et al., 2026). In this framework, *B. luminifera* is proposed to counter nutrient scarcity through two interacting pathways: rhizosphere mobilization of recalcitrant P, and efficient acquisition and internal cycling of nutrients. Because the species-specific evidence base is still incomplete, both pathways are presented here as hypotheses rather than established adaptations, consistent with the evidence-tiering used throughout this review and in **Table 1**.

**Table 1:** Summary of adaptive mechanisms, evidence strength, and key research gaps for *Betula luminifera* in karst habitats.

No.	Adaptive mechanism	Direct evidence	Comparative evidence	Proposed mechanism	Evidence strength	References
1	Efficient photosynthesis-water coupling system	Pn, Tr, WUE data under comparable conditions	Common in karst plants; crown plasticity in heterogeneous light	Trade-off management that prioritizes carbon to photosynthesis and hydraulic integrity under water limitation	High	Fan et al., 2010; Yang et al., 2009
2	High nutrient-use efficiency; efficient internal nutrient cycling	NUE indicators; low C/N, C/P patterns	Common in karst woody plants	Internal reallocation/retranslocation efficiency as cost-saving pathway	Medium-High	Gao et al., 2012; Teng et al., 2016; Cai et al., 2021
3	Active rhizosphere nutrient mobilization (core proposed mechanism)	/	Evidenced in other karst species	P mobilization via exudation, acidification; microbial/phosphatase activity; exudate validation pending	Low-Medium	Rosenvald et al., 2011; Fu et al., 2024
4	Coordinated antioxidant defense; osmotic adjustment	Responses under high-Ca, drought and metal stress	Core strategy in karst plants under Ca/drought stress	Cost-sensitive regulation network; molecular basis of defense-growth trade-offs	High	Tang, 2015; Jiang, 2024; Zhao et al., 2024b
5	Rhizospheric Ca^2+^ retention; root sequestration	/	Sb retention (TF ≪ 1); Widely reported in calcareous-adapted plants	Ca tracers to verify root sequestration; classify *B. luminifera* or state data insufficiency	Medium	Jiang, 2024; Ji et al., 2009
6	Cellular Ca^2+^ compartmentalization & detoxification	/	Established in model species	vacuolar sequestration via CAX transporters and chelation; transcriptomics/metabolomics under high Ca and functional validation	Low	Wang et al., 2024b
7	Dynamic carbon allocation	/	Inferred from photosynthesis, root-shoot ratio, antioxidant activity and growth responses	Prioritize growth, rhizosphere investment, and defense under water limitation	Low-Medium	—
8	Competition & coexistence with other functional groups	/	/	Metabolic-compensation hypothesis; facilitation in *B. luminifera* dominated communities needs synthesis	Very Low	—

Evidence definitions as given in the revised manuscript: direct evidence, measurements obtained specifically in *B. luminifera*; comparative evidence, findings extrapolated from other *Betula* species, karst plants or analogous stress responses; proposed mechanism, logical inference requiring direct validation in *B. luminifera* respectively.

Comparative studies in other species show that plants increase fine-root length and root tip density to cope with P deficiency (Ostonen et al., 2011; Wu et al., 2022a). In *B. luminifera*, fine roots are predominantly concentrated in the topsoil, where they mediate rhizosphere mobilization and mycorrhizal association, whereas deeper roots mainly serve water acquisition and anchorage rather than nutrient foraging (Wang et al., 2007). This functional segregation between the shallow foraging system and the deep root system is also the key link between morphological adaptation and exudative strategy (Bergmann et al., 2020). An enlarged fine-root surface and a higher root-tip density provide more release sites for organic exudates. Exudation is therefore expected to operate hand-in-hand with fine-root foraging (Wen et al., 2022). Morphology provides the surface area, while active secretion supplies the mobilizing compounds. However, direct evidence linking root morphology to exudation intensity in *B. luminifera* is lacking, and this coupling therefore remains part of the working hypothesis outlined below.

It must be emphasized at the outset that direct, species-specific evidence for the rhizosphere P-mobilization pathway in *B. luminifera* is currently unavailable. The mechanism described here includes organic acid exudation, rhizosphere acidification, microbial stimulation, and acid-phosphatase activity. It should therefore be regarded as a working hypothesis rather than an established adaptation. Testing this hypothesis will require direct exudate profiling and rhizosphere manipulation experiments. Under P deficiency, root exudates (notably organic acids and protons) are assumed to acidify the rhizosphere, dissolve Ca-P complexes, and stimulate microbial activity and acid phosphatase secretion, thereby forming a proposed root-microbe-enzyme cascade that mobilizes occluded P and promotes mineralization of organic nutrients (Wang et al., 2023; Li et al., 2024). Several steps of this cascade have support only from non-karst systems: fine-root foraging under P deficiency in Norway spruce (Ostonen et al., 2011), and rhizosphere acidification in silver birch (Rosenvald et al., 2011) and in *Betula platyphylla* (Fu et al., 2024). Because karst soils differ fundamentally in their high exchangeable Ca^2+^ content (which strongly binds P) and in their shallow, discontinuous profile, the transferability of these findings requires direct confirmation through *in situ* rhizosphere sampling or isotope tracing. Until root exudates are characterized in *B. luminifera*, this entire cascade remains a working hypothesis.

Stable isotope data provide preliminary, indirect evidence consistent with efficient N acquisition and reallocation in *B. luminifera.* Its leaves show a distinct δ^15^N value (approximately -2‰) versus ~0.5‰ for *Q. fabri* and ~1.5‰ for *Q. variabilis*, corroborated by higher leaf N content (24.08 mg/g) (Cai et al., 2023; Wu et al., 2024); surface soil (0–30 cm) under *B. luminifera* stands also shows higher δ^15^N (≈7.66‰) than under *Q. fabri* (6.46‰) and *Q. variabilis* (4.03‰), suggesting a potentially more open soil N cycle and greater N availability in its stands. These patterns accord with an acquisitive growth strategy: relatively low tissue C/N and C/P in actively growing leaves indicate N- and P-rich tissues that sustain rapid photosynthesis, whereas higher C/N and C/P in stems and roots reflect greater structural carbon investment. Because C/N and C/P ratios are indirect rather than direct measures of nutrient-use efficiency, and cannot by themselves distinguish acquired, recycled, or conserved nutrients, they are treated here as indicative, not decisive, of internal cycling efficiency. Direct measurements of resorption efficiency and biomass-based nutrient productivity are therefore needed to confirm this interpretation.

Positive plant-soil feedback. The rhizosphere-activation strategy is expected to initiate a positive feedback loop: enhanced nutrient uptake supports growth, leading to increased belowground carbon investment (root turnover, exudation, litterfall). *B. luminifera* litter measurably improves soil fertility. In a 12-year-old plantation, the litter layer stored 108.64 kg/hm^2^ of nutrients (N: 95.50, P: 5.90, K: 7.25 kg/hm^2^) (Zhao et al., 2025), and decomposition experiments have directly demonstrated that its leaf litter significantly raises soil organic carbon, total nitrogen, total phosphorus, and available potassium (Liu et al., 2023). These effects are consistent with ecosystem engineering. However, whether litter-driven fertility changes directly facilitate the establishment of other species, and their long-term community consequences, remain unresolved and require direct successional and facilitation studies.

In summary, the nutrient strategy of *B. luminifera* combines a morphological acquisitive component (a topsoil-dominant fine-root system), a physiological component (proposed rhizosphere P mobilization and an indirect-signaled internal cycling), and a biological component (litter-driven positive feedback). Because direct experimental evidence for the rhizosphere P-mobilization pathway is categorically lacking, this study explicitly classifies the pathway as a proposed mechanism in **Table 1** rather than a confirmed one; the verdict is left pending high-quality data from exudate profiling, *in situ* rhizosphere sampling, and resorption experiments, which could either elevate or discard this hypothesis.

## 4 Integrated ion management and oxidative homeostasis under elevated soil Ca and associated edaphic constraints

High soil Ca is a salient feature of karst environments, yet we explicitly distinguish between the ecological constraints imposed by Ca-rich calcareous soils and the physiological toxicity induced by elevated cytosolic Ca^2+^. For resistant plants, the high total Ca pool is rarely a direct toxicant (Wei et al., 2018); it primarily acts as an indirect environmental filter by (1) precipitating and immobilizing plant-available P (Qetrani et al., 2024), (2) elevating soil pH and inducing associated micronutrient imbalance, and (3) altering water acquisition via changes in soil structure and moisture retention in shallow rocky substrates (Ding et al., 2026). A direct Ca^2+^ toxicity pathway, in which soluble Ca^2+^ exceeds cellular homeostatic capacity, operates mainly under experimentally induced high-Ca conditions. However, for *B. luminifera* in field soils, this pathway remains to be demonstrated.

Before invoking Ca^2+^-specific detoxification, it is therefore necessary to assess whether high Ca directly harms *B. luminifera* or operates primarily through co-occurring constraints. Karst soils are simultaneously characterized by high Ca, alkaline pH, and severe drought, and we cannot yet disentangle their relative causal weights. Consequently, the ion-management mechanisms proposed below (e.g., vacuolar sequestration by CAX transporters, root retention) are candidate responses to high soil Ca whose deployment under natural calcareous soils may equally reflect an integrated adaptation to the complex environment.

We hypothesize that *B. luminifera* maintains homeostasis through two interlinked defense layers. The first layer, ion management, aims to prevent toxicity, with root-level retention restricting the translocation of toxic ions to aerial parts. It must be stressed, however, that the supporting evidence is comparative rather than direct.

The strong root-accumulation pattern with a low translocation factor (TF << 1) was observed under severe Sb contamination (Jiang, 2024). Because Sb is a xenobiotic directly toxic to cellular thiols, such a response cannot by itself demonstrate Ca toxicity; it may, however, reflect a shared ion-management or general stress-response mechanism. Root retention is therefore an inferred, not proven, defense against Ca. This root-sink behavior is functionally analogous to the ion-exclusion strategy of many karst calcicole species (Ji et al., 2009), and indirectly supports, though it does not confirm, a calcicole-type classification of *B. luminifera.* Available tissue Ca data are currently insufficient for a definitive classification, and direct measurements of tissue Ca (and its soluble:insoluble forms) in *B. luminifera* from calcareous soils are needed to resolve its position among calcicole, calcifuge, or indifferent categories. At the cellular level, we further hypothesize that *B. luminifera* manages intracellular Ca^2+^ through mechanisms common to metal-tolerant plants (e.g., vacuolar sequestration and chelation by phytochelatins/metallothioneins); this inference derives from model species rather than direct measurements of vacuolar Ca^2+^ distribution in *B. luminifera*.

The second layer comprises antioxidant and osmotic adjustment to repair stress-induced damage. When cytosolic Ca^2+^ exceeds homeostatic capacity, high Ca^2+^ can disrupt cellular metabolism and induce ROS accumulation with oxidative damage, such as elevated MDA (Liu et al., 2011). This condition, however, has not yet been demonstrated in *B. luminifera* in field soils. *B. luminifera* responds to antimony (Sb) stress through a synergistic enzymatic and non-enzymatic antioxidant system. In leaves, SOD and POD activities are significantly elevated relative to the sympatric species *Symplota tetrogona* (Tang, 2015). However, the deployment is organ-specific. Under Sb stress, SOD increases in leaves, whereas it decreases in roots, where POD and glutathione become the dominant players (Jiang, 2024; Li et al., 2024). It also accumulates low-molecular-weight compatible solutes (proline, soluble sugars) that act both as osmolytes maintaining turgor and as antioxidants stabilizing proteins and membranes (Zhao et al., 2016). Together, these layers reflect active management of ionic and oxidative state rather than passive tolerance, forming the physiological basis for *B. luminifera’s* resilience on karst soils and its value in stabilizing degraded, high-pH sites. As with the Sb-based evidence above, parts of the antioxidant and osmolyte data originate from non-karst (mine-site and laboratory) conditions; their transferability to karst stress combinations requires site-specific validation.

Validating these proposed pathways is a critical next step. Ca^2+^-specific fluorescent probes, subcellular fractionation, and transcriptomic analysis under controlled high Ca conditions (Xing et al., 2026) could directly test whether root retention, vacuolar sequestration, and chelation are indeed deployed against Ca^2+^ stress in *B. luminifera*. Consistent with this hypothesis, this species exhibits strong rhizosphere effects (Lu et al., 2012). Under analogous stresses, it further shows root metal accumulation and upregulation of chelation-related genes such as heat shock proteins (Jiang, 2024). Taken together, these lines of evidence sit on the same comparative level as the root retention described above.

## 5 Integration and trade-offs of carbon as the universal currency under multiple stresses

We emphasize at the outset that the concept of carbon as a “universal currency” allocated among growth, resource acquisition, and defense is presented as an integrative hypothesis or conceptual model, not as a directly verified mechanism in *B. luminifera*. The supporting evidence is indirect. Correlated physiological responses are consistent with carbon reallocation but do not demonstrate it, including elevated antioxidant activity, altered root-to-shoot ratios, and reduced growth. Direct measurements of allocation (e.g., ^13^C/^14^C pulse-chase labeling or non-structural carbohydrate flux tracking) have not yet been performed in this species; isotopic tracing across factorial drought × P × Ca treatments is therefore required to validate the proposed hierarchy.

Conceptually, this framework follows the principles of carbon resource economics (Hu et al., 2022). Photosynthetic carbon is hypothesized to serve as the fundamental resource, or universal currency, that must be dynamically partitioned among the competing demands of growth, resource acquisition, and defense (Herms and Mattson, 1992). Species success thus hinges on the plastic regulation of this partitioning, a process that inherently involves strategic trade-offs. This regulation forms a continuous “acquisition-investment-consumption” cycle that determines fitness under fluctuating stress (Cheng et al., 2021).

Building on the physiological evidence presented in Sections 2–4 and consistent with the growth-defense trade-off framework (Herms and Mattson, 1992; Monson et al., 2022), we propose a tentative carbon-priority hierarchy for *B. luminifera*. At the base of this hierarchy lies the maintenance of hydraulic integrity and basal metabolism. A minimum carbon pool must be reserved for respiration and xylem function, because compromising these would jeopardize survival regardless of subsequent investments. Above this non-negotiable baseline, carbon is preferentially allocated to photosynthetic tissues; the high specific leaf area and elevated water use efficiency (Section 2) indicate that investment in foliar tissues yields immediate returns in carbon fixation, making leaf production a high-return strategy that is hypothesized to take precedence over belowground exudation or secondary metabolism when water is moderately limiting. Beyond these two priorities, carbon follows a cost-sensitive gradient. Under mild stress, growth is favored over osmotic adjustment and root exudation (Huot et al., 2014). This pattern is consistent with the growth-priority carbon allocation described in Section 2. Root exudation for phosphorus mobilization (Section 3) appears to be a lower-priority sink. It is activated primarily under phosphorus deficiency rather than under drought. Defense investment is hierarchically nested rather than sequentially activated. Enzymatic antioxidant responses are constitutive. Secondary metabolite synthesis and vacuolar sequestration are hypothesized to be up-regulated only when stress exceeds thresholds that threaten survival (Monson et al., 2022). It is important to emphasize that this hierarchy is a deductive framework based on the observed trade-offs reported in Sections 2–4, not a directly measured carbon-flux hierarchy; the isotope-tracing experiments described at the outset are required to validate the ordering proposed here.

Available observations are consistent with this hierarchy but remain indirect. Under drought, *B. luminifera* increases its root-to-shoot ratio (Liu et al., 2023). It directs carbon belowground for root construction at the expense of aboveground growth. This pattern is consistent with the first priority of the hierarchy proposed above. In this hierarchy, securing water uptake capacity precedes leaf expansion under water limitation. Exudation for nutrient mobilization and secondary defense remain lower-priority sinks. Exudation is up-regulated mainly under phosphorus deficiency.

This plasticity operates as a precisely regulated, multi-level process. Spatially, *B. luminifera r*etains heavy metals such as Sb in roots to limit shoot exposure while concentrating antioxidant defenses in leaves (Jiang, 2024). Temporally, responses follow a cost-efficient sequence. Low-cost physical barriers are engaged first. More expensive enzymatic synthesis is activated only if stress persists. The stress regime ultimately dictates the balance: under mild or single stress, the systems act synergistically, whereas under severe or compound stress, antagonistic trade-offs dominate and force explicit choices between survival and growth.

Taken together, this chapter integrates the preceding systems through the lens of carbon partitioning: the photosynthetic-water system (Section 2) supplies the currency, rhizosphere mobilization (Section 3) and the defense system (Section 4) compete for its expenditure, and the balance is mediated by a prioritized allocation hierarchy. This hierarchy remains a testable hypothesis rather than an established mechanism, and its validation through isotope tracing and carbon budget modeling under combined stress will be essential to confirming the adaptive value of the proposed framework for karst restoration.

## 6 Individual-level plasticity in resource optimization under environmental filtering

As a light-demanding pioneer, *B. luminifera* exhibits pronounced architectural plasticity along light gradients. In open, high-light environments, it develops expansive crowns to maximize light capture (Li et al., 2023) under shaded conditions, it instead reduces crown length and spread, producing a more compact architecture that minimizes construction costs under light limitation (Yang, 2017). This crown adjustment is complemented by a strategic vertical biomass distribution, with greater investment in the upper and lower canopy layers and reduced investment in the middle to optimize light interception (Yang et al., 2009). At the branch level, shaded individuals tend to proliferate finer secondary branches in the upper canopy rather than reinforcing individual branch strength, an arrangement interpreted as efficiently expanding the light-harvesting surface.

This plasticity operates as a precisely regulated, multi-level process. Spatially, *B. luminifera* retains heavy metals such as Sb in roots to limit shoot exposure while concentrating antioxidant defenses in leaves (Jiang, 2024). Temporally, responses follow a cost-efficient sequence. Low-cost physical barriers are engaged first, and more expensive enzymatic synthesis is activated only if the stress persists. The stress regime plays a decisive role: under mild or single stress, the different systems act synergistically; under severe or combined stress, antagonistic trade-offs predominate and force explicit choices between survival and growth. Although field gas-exchange and antioxidant data support separate drought-photosynthesis and drought-defense interactions, direct experimental evidence for the simultaneous combination of drought, nutrient limitation, and Ca-rich conditions remains scarce. This interaction should therefore be treated as a plausible but as yet unverified consequence of the carbon budget model.

This high degree of allocation plasticity underpins the “high-investment, high-return” strategy of *B. luminifera* (Ning et al., 2011; Gao et al., 2014). In a favorable window, the species prioritizes carbon investment in photosynthetic tissues and rapid vertical growth, contrasting sharply with the conservative strategy of *Q. fabri* (Xing et al., 2015). However, this strategy carries an inherent vulnerability. When extreme stress constricts the available carbon budget, the high metabolic demand cannot be sustained. This places *B. luminifera* at a competitive growth advantage relative to conservative species (Yang et al., 2010). This mechanism plausibly explains its eventual displacement in stable late-successional stages (Zhao et al., 2016). This cost side of plasticity is frequently overlooked in restoration-focused reviews and is explicitly retained here to balance the adaptive narrative.

These plastic traits scale up jointly to define the pioneer function of *B. luminifera* at the community level. Rapid height growth together with flexible crown architecture grants it superior competitive performance for light in open degraded sites (Li et al., 2023). By quickly forming a canopy, it suppresses light-demanding weeds. It also creates shaded microenvironments. These conditions facilitate the establishment of more shade-tolerant species, thereby catalyzing secondary succession (Zhong et al., 2018; Li et al., 2025). In parallel, its stable belowground investment promotes community stability. Persistent root biomass helps stabilize soil structure, and litter input initiates soil-development processes that foster biodiversity and enhance the functional resilience of early-stage communities (Guo et al., 2011). Whether such effects constitute genuine ecosystem engineering, however, rests on the same species-specific evidence needed to treat the underlying mechanisms as established rather than hypothetical.

In summary, individual-level plasticity constitutes the implementation layer of the carbon-priority framework proposed in Section 5, converting the strategic carbon budget into morphological and physiological adjustments along environmental gradients (this section) and, from there into community level consequences. The evidence quality is not uniform across the mechanisms reviewed so far: some have direct experimental or field support, whereas others remain plausible but unverified hypotheses, a distinction summarized together with the critical gaps in **Table 1**.

## 7 Ecological application potential and future research perspectives

The multidimensional adaptive strategies of *B. luminifera* translate directly into substantial ecological application value. They position it as a priority pioneer species for the rehabilitation of diverse degraded ecosystems, including karst rocky desertification, heavy metal-contaminated sites, and severely nutrient-depleted soils. However, its effective deployment in precision ecological restoration ultimately depends on resolving the critical knowledge gaps identified below. These gaps concern the underlying molecular mechanisms, in the multi-scale integration of trait-based effects (Xing et al., 2024, 2026), and in long-term restoration efficacy under ongoing climate change.

This application value is the practical extension of the intrinsic physiological-ecological mechanisms of *B. luminifera* to the community scale, enabling a transition from merely tolerating stress to actively ameliorating site conditions. To provide a systematic overview of the current evidence, we summarize in **Table 1** the key adaptive mechanisms, the strength of the supporting evidence, and the critical knowledge gaps that remain.

This framework evidence linkage is essential because the evidence base is not uniform across mechanisms. Some mechanisms have direct experimental support, whereas others remain plausible but untested hypotheses. This distinction made explicit in **Table 1**. Consistent with this classification, several key mechanisms are presented as hypotheses requiring direct experimental validation. These include rhizosphere phosphorus mobilization via root exudation and calcium (Ca^2+^) management pathways.

A key question for restoration practice is whether *B. luminifera* can accomplish initial ecological rehabilitation in monoculture stands, or whether companion species are functionally indispensable during early-stage recovery. The available evidence does not yet permit a definitive answer: pure plantations can establish successfully on suitable sites, whereas mixed-community trials suggest functional complementarity (Jiang, 2024). However, these datasets derive from different experimental contexts and cannot be directly compared. We therefore identify the monoculture-versus-mixture question as a critical unresolved issue and propose long-term factorial field trials comparing monocultures against species mixtures across degradation gradients as a research priority.

In a five-year mixed-plantation trial in Nanping, Fujian, mixed stands of *B. luminifera* and *Cunninghamia lanceolata* maintained soil fertility, promoted the growth of *C. lanceolata*, and developed a more favorable stand structure than either monoculture, confirming direct interspecific facilitation beyond additive biomass accumulation. Similarly, in a 10-year-old mixed stand of *B. luminifera* and *Populus nigra* var. italica in a karst urban forest, total ecosystem biomass reached 16.70 t ha^−1^, with the tree layer contributing 24.13% of the total (Ning et al., 2011). These observations, although informative, involve contexts and species combinations that differ from natural karst degradation gradients, and their transferability to karst restoration requires direct testing.

Restoration deployment should also account for the complementary functions of the two root compartments of *B. luminifera*: deep roots buffer water stress by accessing fissure water, whereas surface fine roots capture nutrients from the organic-rich layer and stabilize topsoil structure. The root length density data presented in this review quantify the response of the surface fine-root compartment specifically and should not be extrapolated to the deep-root compartment, whose drought-buffering function operates on longer timescales and requires separate verification.

Two caveats on the transferability of this evidence base should be noted. First, most community-level evidence cited above derives from non-karst habitats. Specifically, it comes from heavy metal-contaminated sites, including antimony and lead/zinc mining areas, and managed plantation systems. These contexts differ from natural karst ecosystems in functionally relevant aspects. Contaminated soils impose additional toxic metal stress. They often exhibit altered pH and organic-matter dynamics. They may also host distinct rhizosphere microbial communities. These communities could either amplify or mask the facilitative effects attributed to *B. luminifera*. Second, the outcome of *B. luminifera* based restoration under realistic climate change remains largely untested. This includes extreme drought events and altered precipitation regimes. Long-term observations are required before the species can be recommended across the full gradient of degraded karst sites.

Current research on *B. luminifera* remains fragmented. It focuses largely on isolated traits rather than integrated mechanisms. For instance, stand-level biomass accumulation (Ning et al., 2011; Teng et al., 2016) and species-level stress physiology (Hu et al., 2024) have been examined in parallel. There is little cross-scale synthesis. Moreover, the synergistic effects of multiple simultaneous stressors are poorly understood, and this gap limits the evidence base for precision restoration (Li et al., 2001; Yang et al., 2024). To close these gaps, we identify three priority research directions.

First, molecular and mechanistic scale, the molecular and epigenetic foundations of the key adaptations of *B. luminifera* remain largely unexplored and require direct experimental validation. Critical gaps include the mechanisms underlying high calcium tolerance. These especially concern the proposed root retention and cellular vacuolar sequestration pathways under calcareous conditions. Additional gaps concern the active rhizosphere nutrient mobilization processes, such as the proposed organic-acid exudation for phosphorus activation (Xing et al., 2024, 2026). Addressing these gaps is essential for translating physiological understanding into targeted genetic improvement or silvicultural optimization.

Second, at the community and ecosystem scale, bridging the functional traits of individual trees to ecosystem outcomes requires quantitative characterization of plant-soil feedbacks and interspecific interactions. We identify whether *B. luminifera* exerts net positive or net negative effects on co-occurring species during early restoration as the central question; answering it requires pairwise and community-level manipulative experiments, including leaf-litter removal/addition and species-removal designs that directly test whether the observed soil amelioration causally drives community succession rather than merely co-occurring with it.

Third, at the long-term and applied scale, long-term field trials are essential to evaluate the persistence and resilience of *B. luminifera* based restoration under realistic climate change scenarios, including its capacity to stabilize site conditions and support successive community development, ensuring that the species plays an ecologically sound and sustainable role in karst restoration.
